# "Turn on" and label-free core−shell Ag@SiO_2_ nanoparticles-based metal-enhanced fluorescent (MEF) aptasensor for Hg^2+^

**DOI:** 10.1038/srep09451

**Published:** 2015-03-30

**Authors:** Yuanfeng Pang, Zhen Rong, Rui Xiao, Shengqi Wang

**Affiliations:** 1 Beijing Institute of Radiation Medicine, 27 Taiping Road, Beijing, China

## Abstract

A turn on and label-free fluorescent apasensor for Hg^2+^ with high sensitivity and selectivity has been demonstrated in this report. Firstly, core−shell Ag@SiO_2_ nanoparticles (NPs) were synthetized as a Metal-Enhanced Fluorescent (MEF) substrate, T-rich DNA aptamers were immobilized on the surface of Ag@SiO_2_ NPs and thiazole orange (TO) was selected as fluorescent reporter. After Hg^2+^ was added to the aptamer-Ag@SiO_2_ NPs and TO mixture buffer solution, the aptamer strand can bind Hg^2+^ to form T-Hg^2+^-T complex with a hairpin structure which TO can insert into. When clamped by the nucleic acid bases, the fluorescence quanta yield of TO will be increased under laser excitation and emitted a fluorescence emission. Furthermore, the fluorescence emission can be amplified largely by the MEF effect of the Ag@SiO_2_ NPs. The whole experiment can be finished within 30 min and the limit of detection is 0.33 nM even with interference by high concentrations of other metal ions. Finally, the sensor was applied for detecting Hg^2+^ in different real water samples with satisfying recoveries over 94%.

Mercury is a highly toxic environmental pollutant which distributed in the air, water, and soil widely^1^. The accumulative toxicological effects^2^,^3^,^4^ can harm the brain, heart, kidneys, lungs, and immune system of people at all ages^5^. Water-soluble divalent mercuric ion (Hg^2+^) is one of the most usual and stable form of mercury pollution^6^. Therefore, environmental monitoring of aqueous Hg^2+^ becomes an increasing demand.

Traditional methods for Hg^2+^ quantification include atomic absorption/emission spectroscopy, selective cold vapor atomic fluorescence spectrometry, inductively coupled plasma mass spectrometry (ICPMS). These methods are very sensitive and selective but require complicated sample preparation and sophisticated instruments which limit their application in routine Hg^2+^ monitoring^7^,^8^,^9^. Thus, it is still of great challenge to develop a method which is sensitive, selective, and environmentally friendly for aqueous Hg^2+^ detection. Recently, much effort has been focused on the design of DNA-based sensors to detect metal ions based on the ability of some metal ions that selectively bind to some bases to form stable metal-mediated DNA duplexes^10^,^11^. It has been reported that Hg^2+^ can selectively link T−T pairs to form T−Hg^2+^−T base pairs with high stablity^12^,^13^,^14^. This Hg^2+^-mediated T-T base pair is able to direct the folding of single-stranded DNAs into duplexes or G-quadruplex activate DNAzymes so that it provides a rationale for the utilization of T-containing DNA sequences to specifically sense aqueous Hg^2+^^15^,^16^. A large number of sensors based on fluorescent, colorimetric, and electrochemical sensing have been designed for Hg^2+^ by using the T−Hg^2+^−T coordination chemistry^17^,^18^,^19^,^20^,^21^,^22^. During which, fluorescent methods have attracted great attention because of their distinctive advantages such as simplicity and sensitivity. However, there are still several drawbacks for these sensors: 1. Most of the fluorescent sensors reported work in a “turn-off” mode^23^,^24^,^25^ for which false positive results may be given by other quenchers in real samples. 2. Most of the existing fluorescent Hg^2+^ aptasensor required fluorophores or quencher labeling to the oligonucleotide probes^26^,^27^, which was cost- and time-consuming and increases the complexity. Recently, Cao and Sun group has reported a label-free fluorescent probe^28^ for Hg^2+^ by directly added the signal fluorophores and quencher molecules into the solution based on the fluorescence resonance energy transfer (FRET) signals. However, the fluorescence signals will be affected by the fluorophores or quencher molecule interaction to the T−Hg^2+^−T complex so that the repeatability of this sensor was poor without rigorous experiment conditions control. Zeng group has reported a turn-on and DNA functionalized quantum dots (QDs) fluorescent sensor^29^ for Hg^2+^, however, to realize the Förster Resonance Energy Transfer of QDs, oligonucleotide-Au nanoparticles were also required which would increase not only the complexity of the sensing systems, but also affect the detection sensitivity and selectivity.

In this work, we proposed a label-free and turn-on fluorescent sensor for Hg^2+^ via coreshell Ag@SiO_2_ nanoparticles-based Metal-Enhanced Fluorescence (MEF). MEF is an emerging technology that is expected to be extensively used in fluorescence-based applications in biomedicine and biotechnology^30^. MEF depends on the interactions of the excited-state fluorophores with the surface plasmon resonance of metal particles^31^. Aside from the reduced lifetime for the fluorophore on the metal nanoparticle (NP), the NP is capable of enhancing the local electromagnetic radiation field near the emitter, increasing the possibility of its excitation. With the surface plasmon resonance of metal particles, the quantum yield of fluorophores around the metal particles in the suitable distance can be enhanced to 7–10^9^.

In this study, we prepared coreshell Ag@SiO_2_ nanoparticles as the MEF substrate and expected it to increase the sensitivity for the Hg^2+^ detection based on the aptasensor system. The sensing mechanism is illustrated in Fig. 1. Initially, a coreshell Ag@SiO_2_ nanoparticle with an Ag NP core and a silica shell spacer of precise thickness was synthesized and the T-rich ss-DNA aptamer was immobilized onto the surface of Ag@SiO_2_ NPs, the thiazole orange (TO) was chosen as a fluorescent reporter. In the absence of Hg^2+^, aptamers kept the single strand structure and TO was free in the solution, almost no fluorescence emission can be detected under laser excitation^32^. However, after adding Hg^2+^, the single strand fold to hairpin duplex structure due to the T-Hg^2+^-T combining. In that case, TO could insert into the hairpin complex and clamped by the nucleic acid bases with higher fluorescence quantum yield when excited by a laser. Meanwhile, the fluorescence emission of the excited-state TO was further amplified by the MEF effect of Ag@SiO_2_ NPs, so that the sensitivity of the aptasensor can be increased largely. To our best knowledge, this is the first label-free MEF-based aptasensor for heavy metal ions detection.

## Results

### Characterization of the Ag@SiO_2_ NPs and aptamer-Ag@SiO_2_ NPs

**Figure 2:**
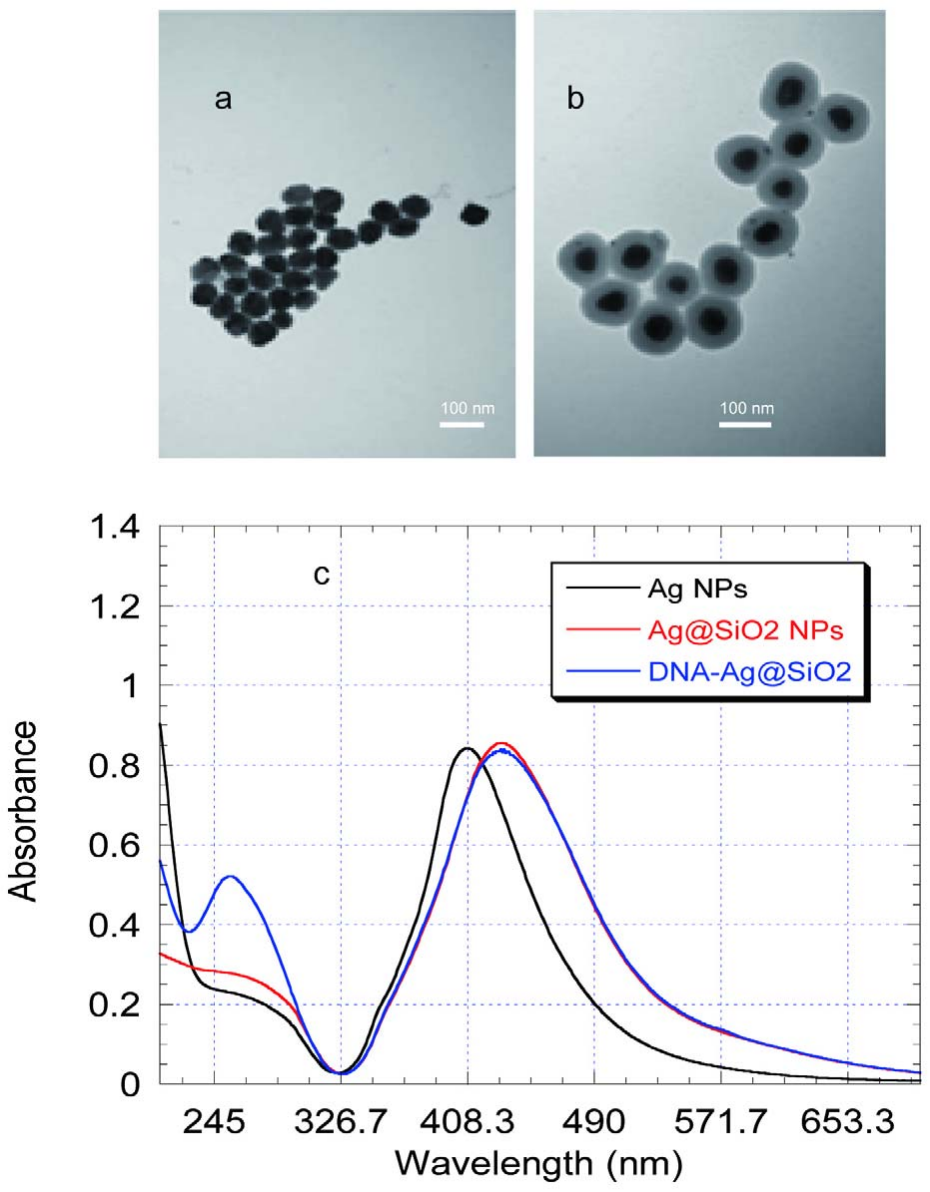
(a) TEM images of the prepared Ag NPs with an average diameter of 50 ± 2 nm and (b) coreshell Ag@SiO_2_ NPs with 30 ± 2 nm shell thickness. (c) UV-vis spectra of Ag NPs, Ag@SiO_2_, and Ag@SiO_2_ immobilized with DNA aptamer. The average diameter and standard deviation were caculated from 100 Ag@SiO_2_ NPs of the same batch by independent measurements.

Firstly, the characterization of the Ag@SiO_2_ NPs and DNA aptamer immobilization were verified. Fig. 2a and 2b show the TEM morphology of the Ag NPs and Ag@SiO_2_ NP s respectively. From Fig. 2a, the Ag NPs showed an average diameter at 50 ± 2 nm (The average diameter and standard deviation were calculated from 100 Ag@SiO_2_ NPs of the same batch by independent measurements.), thus the Ag NPs can be expected to indicate highly enhanced fluorescence compared with which reported in the previous paper^33^. Fig. 2b showed the Ag NPs were coated with a silica shell and the silica shell thickness was about 30 ± 2 nm (The silica shell thickness can be adjusted by changing the amount of reagents mentioned in the Ag@SiO_2_ NPs preparation section). Fig. 2c showed the UV absorbance spectra of the bare Ag NPs, Ag@SiO_2_ NPs with and without DNA aptamers modified. Compared with the bare Ag NPs(Fig. 2c, curve 1), Ag@SiO_2_ NPs (Fig. 2c, curve 2) showed a red shift of 21 nm (from 408 to 429 nm) in the plasmon resonance band which was induced by the increase in the local refractive index of the silica shell and the decrease in plasmon oscillation energy, there was an obvious absorbance at 260 nm after DNA aptamers immobilization which proved that the DNA had been immobilized on the surface of the Ag@SiO_2_ NPs successfully (Fig. 2c, curve 3). The coverage density was calculated to be 502 ± 21 DNA for each Ag@SiO_2_ NP.

### Verify the superiority of the Ag@SiO_2_ NPs-based MEF biosensor

**Figure 3:**
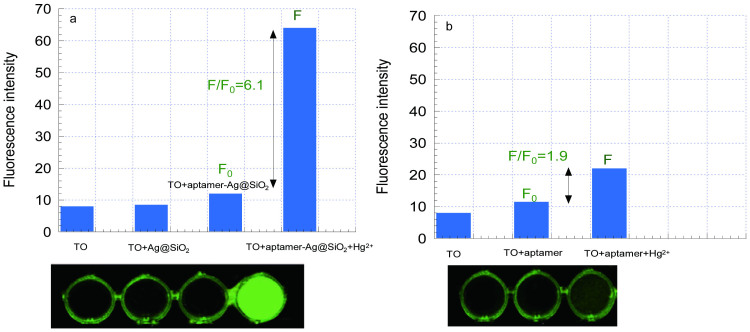
(a) Fluresence intensity of TO (400 nM) alone in the buffer solution; TO mixed with the Ag@SiO_2_ NPs (0.5 nM); TO mixed with aptamer-Ag@SiO_2_ NPs (0.5 nM) with (F) or without (F_0_) Hg^2+^ (1000 nM) added. (b) Fluresence intensity of TO (400 nM) alone in the buffer solution, TO mixed with the aptamer (100 nM) with (F) or without (F_0_) Hg^2+^ (1000 nM) added. Pictures were obtained by using a luminescent image analyzer with a charge-coupled device camera (LAS-1000 mini, Fujifilm Corp., Tokyo, Japan).

To verify the superiority of the Ag@SiO_2_ MEF sensor, the Hg^2+^ detection by aptamer alone and by aptamer-modified Ag@SiO_2_ NPs at the same other conditions (in the buffer solution with TO 400 nM) was operated and the result was shown in Fig. 3. To show the fluorescence intensity difference directly, fluorescent image pictures were displayed under the fluorescent data graph by using a luminescent image analyzer with a charge-coupled device camera (LAS-1000 mini, Fujifilm Corp., Tokyo, Japan). Fluorescence intensities are displayed.

From Fig. 3a, the fluorescence intensity TO (400 nM) alone in the buffer solution was very small and there was almost no difference after TO mixed with the Ag@SiO_2_ solution, that is because in the absence of target Hg^2+^, TO kept in an free state with a negligible fluorescence emission under laser excitation, so the MEF effect of Ag@SiO_2_ NPs to TO free in the solution can be ignored. It is a merit for a turn on sensor because the Ag@SiO_2_ NPs would not increase the background signal and only showed MEF effect after target added, the low background signal will increase the detection sensitivity. The fluoresence intensity changed little when TO mixed with aptamer-Ag@SiO_2_ (0.5 nM) solution, however, after Hg^2+^ (1000 nM)was added into the mixture solution,the fluoresence intensity increased about 6.1-fold and there was also a obvious fluorescent emission image. For Fig. 3b, there was little increase before and after TO (400 nM) mixed with the aptemers (100 nM), after Hg^2+^ (1000 nM) added, the fluoresence intensity increased about 1.9-fold and the fluorescent emission image was also very weak. These results showed the MEF effect of the Ag@SiO_2_ NPs can really increase the signal-to-noise ratio so that detection sensitivity of the aptasensor can be increased largely.

### Optimization of the assay conditions

**Figure 4:**
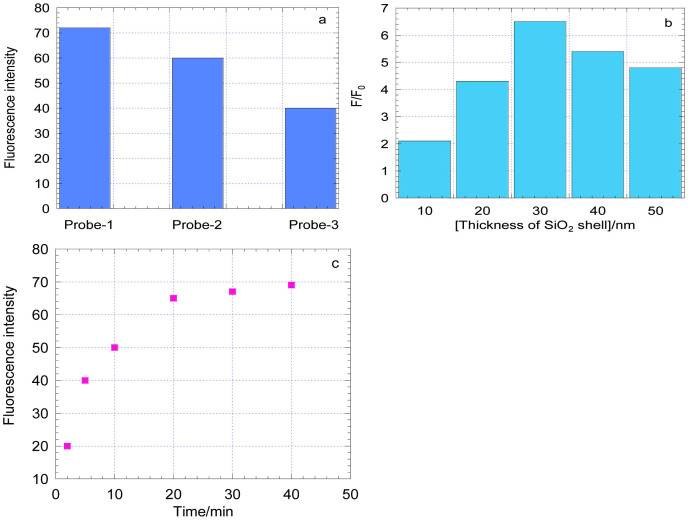
(a) Fluresence intensity of 3 kinds of probe-Ag@SiO_2_ NPs (0.5 nM) in the solution of TO (400 nM) and Hg^2+^ (1000 nM) (b) The fluorescence intensity response F/F_0_ of the system solution to a series of silica shell thicknessand. (c) The fluorescence intensity response F/F_0_ to the incubation time. (F_0_ denoted the fluorescence intensity (538 nm) without Hg^2+^ and F denoted the fluorescence intensity (538 nm) with 1.0 μM Hg^2+^).

For our label-free aptasensor by using TO as signal reporter, an optimal selection of T rich ss-DNA aptamer is pivotal. In this work, three oligonucleotides, with different T base amount and position in the sequence were selected and tested. They are: Probe-1(5'-GTCGTCGTCGTCAAAAGTCGTCGTCGTC-3');probe-2(5'-CGCTGTCTGTCAAAAGTCTGTCTGCG-3') and Probe-3 (5'-GCGCTTTTTTCAAAAGTTTTTTGCGC-3'). In the three probes, T bases were segregated by the CG bases as the fluorescence reporter TO has higher affinity with (CG)_n_ sequence^34^.In probe-1, one T base was segregated by two other bases, in probe-2, T bases were segregated one by one. In probe-3, six T bases were in a continuous state. As illustrated in Fig. 4a, with the addition of Hg^2+^ into different probe-Ag@SiO_2_ solutions (with TO 400 nM), probe-1 showed the highest fluorescence intensity, while probe-3 showed the lowest fluorescence intensity. That may be induced by the stacking interactions of T-Hg^2+^-T complex to TO inserting into the DNA duplex. Another reason was the static electricity excluding effect of the positive charge of Hg^2+^ to the TO molecular which also contained positive charges in the head. Therefore, compact T bases arranging may block the TO binding to the hairpin DNA structure. We also tested the DNA probes for which T bases were segregated by three or more other bases and the fluorescence intensity was also very low, the reason may be that the formation of T-Hg^2+^-T complex was difficult for long interval between T bases. Therefore, the probe-1 was finally selected as the constructing fluorescent aptamer for Hg^2+^ detection.

In the present strategy, the MEF ability of the Ag@SiO_2_ and the incubation times played crucial roles in the detection sensitivity. MEF is affected by numerous factors such as the type of metal NPs, size and morphology of the NPs, spatial distance between the fluorophore and NP surface^35^, and quantum yield of fluorophores^36^. Among these factors, the distance dependence is important in the fluorescence enhancement. Here, a SiO_2_ matrix was used as a shell around the Ag core for distance control and the thickness of the silica shell can be adjustable by simply controlling the amount of chemicals. A series of Ag@SiO_2_ NPs with different silica shell thickness from 10 ± 1 nm to 50 ± 1 nm (1 nm is the RSD of the length meter) was synthetized. The fluorescence enhancement ability was detected by adding the aptamer-modified Ag@SiO_2_ NPs into the buffer solution with Hg^2+^ 1.0 μM and TO 0.4 μM. Fluorescence enhancement factor F/F_0_ (F_0_ denoted the fluorescence intensity (538 nm) without Hg^2+^ and F denoted the fluorescence intensity (538 nm) with 1.0 μM Hg^2+^) can be seen in the Fig. 4b. It proved that in the same other conditions, with the increase of the shell thickness from 10 to 30 nm, fluorescence intensity increased and reached a peak at 30 nm. That may be the combining result of the FRET effect and the Purcell effect^36^,^37^,^38^. For metal nanoparticles (diameter <15 nm), the fluorophore will be excited through FRET by the intense local field of the plasmon (<10 nm). For larger metal nanoparticles greater than 15 nm, an enhancement will be possible through both FRET at close distances (10 nm) and the Purcell effect at longer distances (10–50 nm). Thus, for our Ag@SiO_2_ NP (Ag NP ~50 nm)with the silica shell thickness of 30 nm was chosen. Another factor is the incubating time of Hg^2+^ with our biosensor system. After adding 1.0 μM Hg^2+^ to the sensing system, the fluorescence response with time was recorded and the result was shown in Fig. 4c. It showed that fluorescence intensity increased with increasing incubation time and the highest value was observed at 20 min, and then remained almost constant. Thus, 20 min was used as the optimal incubated time.

### Sensitivity of the sensor for Hg^2+^ detection

**Figure 5:**
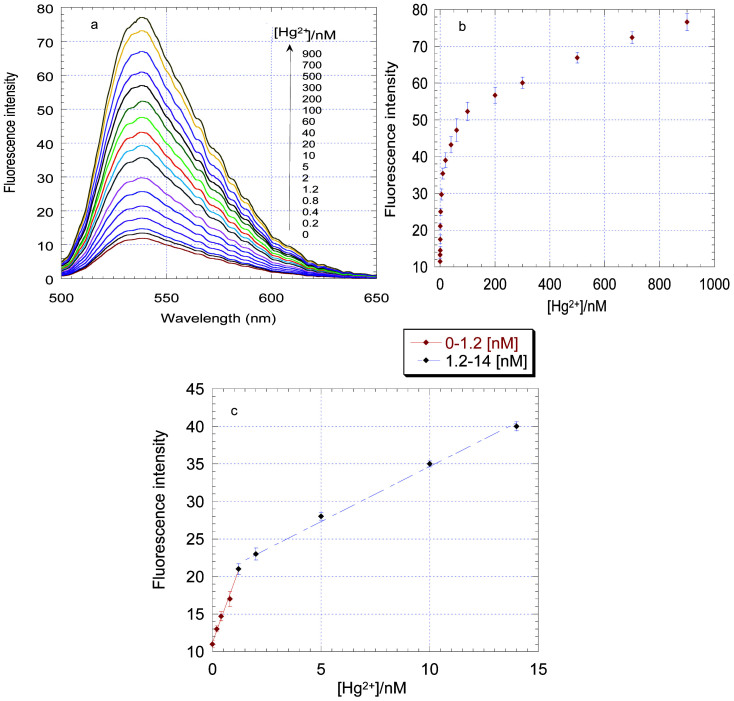
(a) Fluresence spactra and (b) fluoresence intensity response of the aptamer-Ag@SiO_2_ NPs solution (with 400 nM TO) upon addition of Hg^2+^ (0–900 nM). (c) The linear response with Hg^2+^ concentrations from 0 to 1.2 nM and from 1.2 to 14 nM. λ_ex_ = 482 nm, λ_em_ = 538 nm. Error bars represent standard deviation of three independent measurements.

Determination of Hg^2+^ under optimal conditions was operated in buffer firstly. Fig. 5a and 5b showed the fluorescence spectra and intensity (538 nm) increased as the concentration of Hg^2+^ increasing in a dynamic range that spanned 0 to 900 nM. Fig. 5c showed the linear response from 0 to 1.2 nM and from 1.2 to 14 nM. From the slope of the linear regression curve and the standard deviation of the blank signal, the limit of detection (LOD) was estimated to be 0.33 nM. This is an exceptionally low or comparable result compared with other turn-on fluorescent methods for Hg^2+^. For example, the quantum dots and gold nanoparticles fluorescent sensor (0.18 nM)^29^, the [Ru (bpy) _2_(pip)]^2+^ and graphene oxide (GO) system (2.34 nM)^28^, the evanescent wave fiber optic biosensors (1.2 nM)^27^. To the best of our knowledge, this is the first complex of the Ag@SiO_2_ NPs MEF ability and the Hg^2+^ aptasensor. This novel sensor can be applied to the direct detection of Hg^2+^ in natural water with the ability to meet even the most stringent requirements demanded by the USEPA (10 nM). For examination of the repeatability of this sensor, three sample solutions with different concentrations of Hg^2+^ were prepared, and the relative standard deviations (RSDs) are about 5.1%, 3.6%, and 2.3% for three independent determinations of 0.5, 5.0, and 10 nM Hg^2+^ under the optimum conditions, respectively.

### Selectivity of the biosensor system

**Figure 6:**
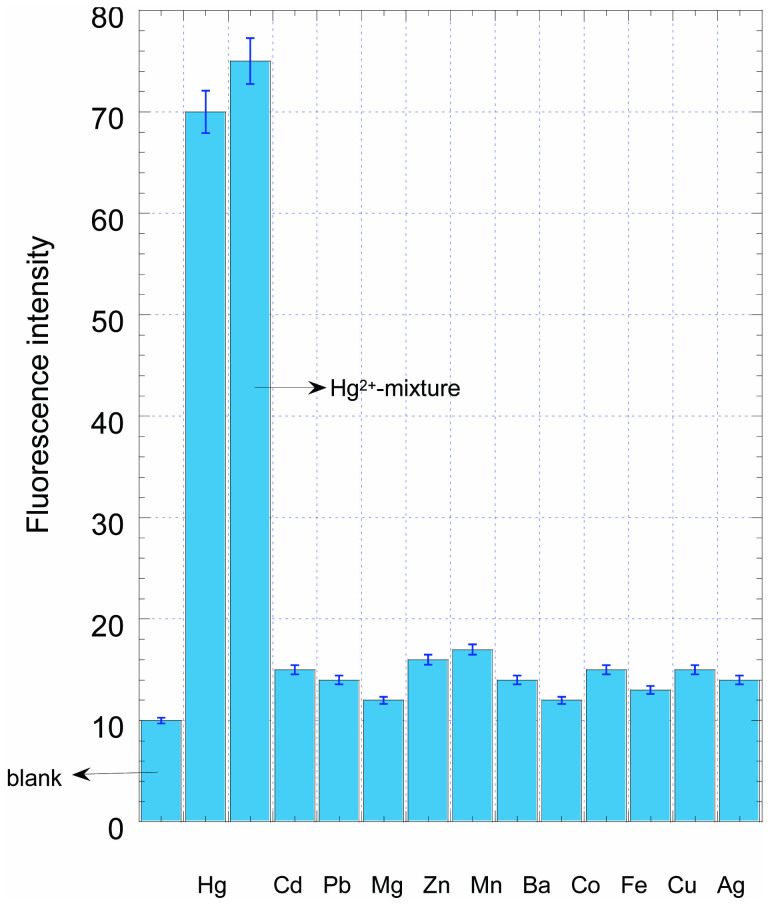
Selectivity of the fluorescent Hg^2+^ sensor. The concentration of Hg^2+^ was 1.0 μM and other metal ion was 10 μM. Every data point is the mean of three measurements. The error bars are the standard deviation.

To demonstrate the selectivity of this sensor toward Hg^2+^, the competitive metal ions, including Cd^2+^, Pb^2+^, Mg^2+^, Zn^2+^, Mn^2+^, Ba^2+^, Co^2+^, Fe^2+^, Cu^2+^ and Ag^+^ were examined under optimized conditions. First, 1.0 μM of Hg^2+^ and 10 μM of other metal ions were added to the sample solution respectively, second, 1.0 μM of Hg^2+^ and 10 μM of other metal ions were mixed together to form a mixture solution as a sample for interference testing. As indicated in Fig. 6, only Hg^2+^ and the Hg^2+^-mixed sample exhibited significant response. These results demonstrated that the compositions of real samples did not significantly interfere with the detection of Hg^2+^, which indicated the potential applications of our method for the analysis of Hg^2+^ in complicated environmental samples.

### Detection of Hg^2+^ concentrations in environmental water samples

To further evaluate the potential application of this sensing system in some real samples, the sensor was tentatively applied to sensing Hg^2+^ in real samples such as tap water and lake water samples. To prove the reliability by our sensor, the same detection was operated by using AFS (atomic fluorescence spectrometer) and the results are summarized in Table 1, with the good agreement between the two methods, it proved that the present sensor can also work in environmental water samples.

## Discussion

Compared with previous works on mercury detection using Hg^2+^-aptamer optical sensors, there are several virtues for our sensor: 1. The cost-consuming and time-consuming fluorophore labeling to the aptamer can be omitted. 2. As a turn-on sensor, the background noise induced by signal reporter tags was very low so that the sensitivity of the fluorescent sensors can be increased. (That is because in the absence of Hg^2+^, TO keep in a free state with low fluorescence quanta yield when excited.) 3. By using the Ag@SiO_2_ NPs as the MEF substrate, distance between the fluorophore and Ag NPs surface can be controlled with nanometer accuracy through adjusting the thickness of the silica shell by simply controlling the amount of chemicals. Therefore, this MEF substrate was universal and can be adjusted to adapt to other fluorescent aptasensor system. 4. The whole detection process only need one kind of DNA strand and the whole process can be finished in a PE tube within 30 min without sample pretreatment and multi-hybridization or washing steps.

In conclusion, we have successfully fabricated a MEF-based fluorescent aptasensor for Hg^2+^, the detection limit was 0.33 nM which was much lower than the EPA limit of Hg^2+^ in drinkable water (<10 nM).Meanwhile, due to the T-T mismatches selective binding to Hg^2+^, this approach is not only insensitive to other metal ions but also selective toward Hg^2+^ in their presence. Therefore, this sensing system exhibits satisfying performances in real environmental water samples.

The Ag@SiO_2_ NPs MEF-based platform can be generally applicable to any aptamer and corresponding biomolecules. Future work will include the application of various types of immobilized DNA probes for small molecules, proteins, metal ions and even whole cells.

## Methods

### Preparation of coreshell Ag@SiO_2_ NPs

Ag nanoparticles with diameter about 50 nm were synthesized in large scale via a modified method reported^39^ Ag@SiO_2_ NPs were synthesized according to the literature^40^. Briefly, Ag colloid solution was mixed with NH_3_·H_2_O (30%) and TEOS (20%) solution to react for 30 min at room temperature and aged at 4°C overnight. The silica-coated Ag NP suspension was washed three times with a water and ethanol mixture (5:4) and centrifuged at 6000 rpm for 20 min and suspended in water, and the thickness of the silica shell was determined by TEM.

### Immobilization of aptamer onto Ag@SiO_2_ NPs

The prepared Ag@SiO_2_ NPs were mixed with 500 μL of 3-APTS and 1 mL 2, 4, 6-trichloro-1, 3, 5-triazine solution (0.2 M) to react for 2 h at room temperature. After centrifuged, it was washed three times with acetonitrile and then dispersed in the phosphate buffered saline (PBS; 0.1 M NaCl, 10 mM phosphate, pH 7.4). Finally, the aptamer (500 μL, 1 μM) were added and incubated for 14 h at 40°C for immobilization, unconjugated aptamers were removed by centrifugation at 10000 rpm for 15 min and repetitive washing with 0.1 M PBS three times.

### Procedures for Hg^2+^ detection

A phosphate buffered saline solution (PBS; 0.1 M NaCl, 10 mM phosphate, pH 7.4) was used for the detection of Hg^2+^ or other metal ions in buffer or real environmental water samples. Various concentrations of Hg^2+^ (20 μL) were added into the aptamer-Ag@SiO_2_ (0.5 nM) and TO (0.4 μΜ) mixture solution and incubated for 20 min to form THg^2+^T coordination chemistry. Finally, the fluorescence spectra of different concentrations of Hg^2+^ were monitored. For the sensitivity experiment, the concentrations of Hg^2+^ were from 0 to 900 nM. For the optimizing experiment, 1.0 μM Hg^2+^, 0.4 μΜ TO and 50 nM aptamer-Ag@SiO_2_ were used to get the optimizing aptamer sequence, silica shell thickness and incubation time. Various metal ions of Cd^2+^, Pb^2+^, Mg^2+^, Zn^2+^, Mn^2+^, Ba^2+^, Co^2+^, Fe^2+^, Cu^2+^ and Ag^+^ of 10 μM were used in the selectivity experiments. Tap water samples obtained from our institute and a lake in the campus of Beijing University (Beijing, China) were filtered through a 0.2 μm membrane. The water samples were spiked with standard Hg^2+^ solutions at certain concentrations and then mixed with the buffer solution containing the aptamer-Ag@SiO_2_ NPs and TO. Atomic fluorescence measurements were performed on an atomic fluorescence spectrometer (AFS-9700) (Beijing, China). Transmission electron microscopy (TEM) images were detected with a JEM-2100 transmission electron microscope at with an accelerating voltage of 200 kV. (Akishima, Japan).Absorption spectra were measured with a JASCOV-570 UV/vis spectrophotometer (Tokyo, Japan), fluorescence spectra were measured with a LS-55 spectrofluorometer (Perkin-Elmer, U.S.A.)

## Author Contributions

Y.F.P. designed and performed the experiments and wrote the manuscript. R.Z. synthetized the Ag@SiO_2_ NPs and provided the TEM images. R.X. and S.Q.W. designed and managed the project. All the authors discuss the results and commented on the manuscript.

## Figures and Tables

**Figure 1:**
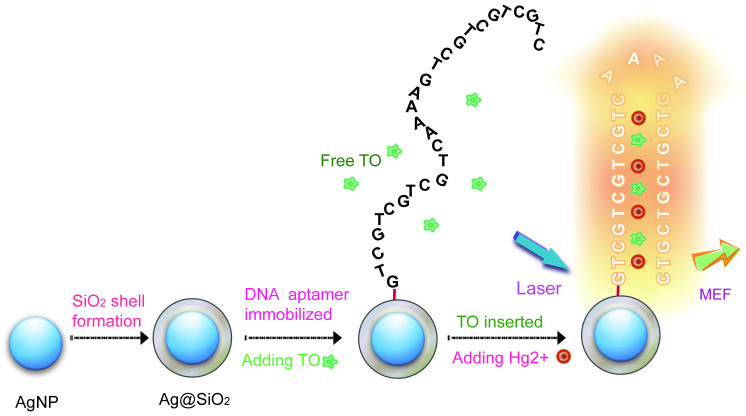
Schematic illustration of the preparation of the MEF-based aptamer-Ag@SiO_2_ sensor and the determination of Hg^2+^.

**Table 1:** Recoveries for the determination of Hg^2+^ in real water samples (n = 3)

Sample(nM)	added	proposed method	recoverry(%)	RSD(%)	AFS ± SD
Tap water	0	N			N
	1.0	1.06	98.5	0.76	1.03 ± 0.008
	2.0	2.05	103.5	1.35	1.98 ± 0.055
	10.0	10.14	101.0	1.22	10.16 ± 0.075
Lake water	0	N			N
	1.0	1.05	94.3	0.78	0.99 ± 0.062
	2.0	2.04	97.2	1.25	2.10 ± 0.010
	10.0	10.11	105.5	1.40	10.09 ± 0.077

RSD = Relative Standard Deviation.

SD = Standard Deviation.

N = no Hg^2+^ was detected.
